# Dr. Blegborough, on Gout

**Published:** 1804-10-01

**Authors:** Ralph Blegborough

**Affiliations:** Margaret Street


### *To the Editors of the Medical and Physical Journal.*

Gentlemen,

It is as easy to know some men by their style and turn of sentiment as by their faces. Few of your Readers, I apprehend, will doubt, nor have I much hesitation in considering my friend, Dr. Kinglake, as the author of the letter signed Candidus, in your last Journal. I trust he will not quote it as evidence in the second edition of his Dissertation on Gout, provided I shall not be able to convince him and the world of the dangerous tendency of his doctrine before a second edition be called for. The Doctor thought it right to advertise for the real signature of E. O. and found him to be a very modest, ingenuous young

 man, man, with, as he declares himself, very little experience. The same step was unnecessary, or I am grossly mistaken, with regard to PERSCRUTATOR; for who wrote that letter, I think, he who runs may read. But the Doctor does not seem very delicate in his selection of materials, in assisting him to make a book: however, as this last letter really does him so much credit on the score of *candour* at least, I am surprised that he should not have thought it right to have had his own name affixed to it. So far are we indebted to the word *liberal* in one of my letters, which I acknowledge to have produced the very effect I intended; and I congratulate the Doctor on his recovery from the influence of that *unlucky* letter of *A Constant Reader,* which gave him so much cruel cause of offence. I hope, in future, he will attempt "*nihil facere per iracundiam;*" as he will now be able to judge dispassionately, how easy it is for intemperate zeal to injure the cause it is intended to support. To make more sure of the matter, however, I shall introduce the Doctor to my friend Leibnitz, who shall tell him a story about a Leyden cobler.

"Quand on disputoit des Théses a l'Université de Leyde, un cordonnier ne manquoit jamais de se trouver a la dispute publique. Enfin, quelqu'un qui le connoissoit lui demanda, s'il entendoit le Latin? *Non,* dit-il, *& je ne veux pas même me donner la peine de l'entendre.* "Pourquoi venez-vous donc si souvent dans cet auditoire, ou l'on ne parle que Latin?" *C'est que je prends plaiser à juger des coups.* "Et comment en jugez-vous sans savoir ce qu'on dit?" *C'est que j'ai un autre moyen de juger, qui a raison.* "Et comment?" *C'est que quand je vois à la mine de quelqu'un qu'il se fache, & qu'il se met en colere, je juge que les raisons lui manquent.*"

But to our subject. The Doctor says, "*All this, it must be confessed, is new, not unplausible, and has a very generalizing tendency.* That it is new, and has a very generalizing tendency I admit; but I also hope that the Doctor will be *candid* enough not to press the other point, when he has been so repeatedly told, that his doctrine is not only very *unplausible,* but very *dangerous* too. A City Alderman, for instance, or a *Common-council-man,* nay, even my Lord Mayor himself, with two pounds of turtle, as much venison, and three or four bottles of claret under his belt, may follow the Doctor's directions if they please; but I hope, nevertheless, I shall prove it unfit for ordinary patients.

I read as much of the DISSERTATION ON GOUT as I could could, with a view to find out, "*that the disease is merely local, and unknown as a constitutional complaint;*" and own I missed it, unless it be contained in the following passage, page 19, "*That various causes connected with temperature, diet, and constitution, operating on systematic excitability, may from accidental preponderance of motive susceptibility on the ligaments and tendons, finally exert a concentrated or inflammatory influence on those parts, and thus induce the formal character of gout!*" I labour to be perspicuous, and really, sometimes at least, flatter myself that I am so; and know not what in my writings can be called "*elaborate and inexplicable suppositions, and theoretic declamation,*" by the author of the above rhapsody. The last and most *plausible* charge, which the Doctor brings against me is, that I profess to cure relaxation by relaxing means. I plead guilty to the charge; and will endeavour to explain this seeming paradox in a few words on this, because I have done it more at large on other occasions. By the application of steam then, and removing a part of the pressure of the atmosphere, I apprehend the paroxysm is often carried off at once along with the disposition in the parts to resist by a fruitless struggle its distensive power, or what the Doctor calls, "*its decomposition of organic structure by combustive force.*" I do not wonder that the Doctor from all his reasoning, should not know, that heat is more easily excited in young than in old subjects, and I suppose that he does not read Mr. Rigby's book, lest he should be convinced of this truth; alas! *nemo scit quam nescit.*

But facts are what the Doctor wants, and perhaps the following, which I am truly sorry at the necessity of recording, will suffice.

To the Rev. J. N. FREEMAN,

*Hayes, near Southall, Middlesex.*

Rev. Sir,

Though an entire stranger to you, I shall make no apology for addressing you on a subject which at present occupies much attention, and nearly concerns the well-being of thousands.

I have heard that Mr. Baker, a medical gentleman, of Uxbridge, died lately after having applied cold water, or ice, to the lower extremities, while labouring under gouty inflammation.

Mr. Reynolds, of Oxford Street, has assured me, that you will consider it a duty you owe society to communicate what you know of this matter; and I trust you will have have no objection to do so to me, who am at present *conscientiously* combating the principle; and that you will not object, provided your statement suit my purpose, to permit me to lay it before the public.

"I am,
"Rev. Sir,
"Your's most obediently,
*August* 21, 1804. "Ralph Blegborough."

To Dr. BLEGBOROUGH,
*Margaret Street, Cavendish Square, London.*

"Sir,

"The Rev. Mr. Freeman, of Hayes, shewed me a letter yesterday from you, which I thought it right to communicate to the family of the gentleman lately deceased; and as they are extremely desirous, that all personal controversy should be avoided, they have requested me to inform you, that no detail of the case can be given to any person.

"Should circumstances, however, occur to induce them to alter their opinion, I propose to communicate it myself to the public, through the medium of the Medical and Physical Journal. "I am,
"Sir,
*Uxbridge,* "Your's, very respectfully,
*August* 24, 1804. "A. Edlin."

"There is a holy mistaken zeal in" Medicine "as well as in Religion. By persuading others we convince ourselves. The passions are engaged, and create a maternal affection in the mind, which forces us to love the cause for which we suffer." Out of tenderness to the friends of the deceased then, and that I may not increase the Doctor's attachment to his opinions by intemperate opposition, I think it most prudent to forego all comments on the above correspondence. In return, I trust, he will not call for more proofs from me; not because I am unprepared to give them, but because I am *tremblingly alive* to the feelings of other families, and other physicians. That such cases have not occurred to himself, can only arise from a contracted sphere of practice. That they may not occur to him in future, I trust he will avail himself of the fell experience of others. After having been prosecutor, counsel, and evidence, in his own cause, the Doctor *candidly* though reluctantly declines being judge, and calls on the public. I am satisfied with, and reverence the tribunal, and am confident of its verdict. I hope, however, I need not not repeat, that I am far from wishing to explode altogether the abstraction of heat from parts labouring under gouty inflammation; for the truth of the matter is, that I do not think my experience, considerable as it has been, as yet sufficient to justify me in giving a decided opinion on a subject of so much importance.

I beg pardon if I have betrayed any warmth during this Controversy, which, I hope, is now brought to a close. In entering into it, I declare solemnly I was actuated by very different motives to what have been attributed to me. I, who am very irritable myself, should be the last person in the world to blame Dr. Kinglake for impetuosity. I am very sensible that, along with every other good thing, we receive our tempers and dispositions also from the Author of our being; and "*that it was He who made us, and not we ourselves.*"

I am, &c.

*Margaret Street, Sept.* 3, 1804. RALPH BLEGBOROUGH.

P.S. As a member of the Committee, I take leave to inform your Bedfordshire Correspondent and Subscriber to Dr. Garnett's work, that it will be out very soon; in all probability, in the course of a month. I would willingly (as well on account of its intrinsic merit, as on behalf of his orphan children) and am sure I can confidently, recommend it to every medical gentleman and philosopher in the united kingdom. Nor do I think I can better close this communication, than with an extract from the Lecture on Gout.

"If the Gout were of a sthenic or inflammatory nature, might we not ask, why the causes which produce it, do not produce it in the meridian of life, when they produce their greatest effect, and when real sthenic diseases are most apt to occur? Or, why the symptoms of the inflammation, like all other sthenic inflammations, are not relieved by the debilitating plan? The contrary, however, points out to us clearly the nature of the disease: the gout is not a sthenic disease, nor a disease of strength; it does not depend on increased vigour of the constitution, and plethora; but is manifestly asthenic, like all the rest of the asthenic diseases. The mode of living is such as brings on indirect debility, or exhaustion of the excitability, such as the use of rich and highly seasoned food, and a daily use of fermented liquors. These, at first, certainly produce vigour, or strength, and will be the cause of sthenic diseases; but they are generally taken in such a manner, that that though they produce a degree of excitement above the point of health, still they only approach the line of sthenic disease, without in general falling into it. They continue, however, to exhaust the excitability, and by the time that the vigour of the body begins naturally to decline, the system of a person who has lived in this manner is unusually torpid. All the blood-vessels, which have been hitherto distended with rich blood, begin to lose their tone, from their excitability having been exhausted by the use of these powerful stimulants; but this torpor is particularly and first experienced in those parts, which have been more immediately subject to the action of the exciting causes, viz. the stomach and bowels: symptoms of indigestion occur, and the excitability of these organs having been almost entirely exhausted by the violent action of the stimulants applied, cannot now be roused to any healthy action; the food is not properly digested, but runs into a kind of fermentation, which causes an extrication of gas; this distends the stomach and bowels, and produces pains, uneasy eructations, and all the distressing symptoms of indigestion. Nor is this in the least surprising, when we consider that many people, who have brought on complaints of this kind, have been in the habit of eating heartily of rich and highly seasoned animal food, and of drinking from a pint to a bottle of wine, and perhaps a quantity of malt liquor, almost every day of their lives, for years. This mode is sufficient to wear out the powers of the stomach, were it three times as capacious as it is; and of the constitution, were it ten times as strong.

"When a torpor, or state of exhausted excitability of the whole system, has been induced in this manner, and symptoms of indigestion produced, any directly debilitating cause applied to the extremities, adding to the indirect debility, causes a total torpor or inactivity of the minute vessels of the part, and thus totally destroys the balance between the propelling and resisting force; hence the vessels will be morbidly distended with blood, a swelling and redness will take place, and an asthenic inflammation produced in the way which I fully pointed out in the last Lecture will be established. Hence the pain and other symptoms which accompany a fit of the gout; hence likewise we see, why debilitating powers, applied to the part, will not reduce the inflammation; and why a warmth, which aggravates every really sthenic inflammatory affection, is so comfortable in this."

